# Trends and opportunities in computable clinical phenotyping: A scoping review

**DOI:** 10.1016/j.jbi.2023.104335

**Published:** 2023-03-16

**Authors:** Ting He, Anas Belouali, Jessica Patricoski, Harold Lehmann, Robert Ball, Valsamo Anagnostou, Kory Kreimeyer, Taxiarchis Botsis

**Affiliations:** a Department of Oncology, The Sidney Kimmel Comprehensive Cancer Center, Johns Hopkins University School of Medicine, Baltimore, MD, USA; b Biomedical Informatics and Data Science Section, Johns Hopkins University School of Medicine, Baltimore, MD, USA; c Office of Surveillance and Epidemiology, Center for Drug Evaluation and Research, US FDA, Silver Spring, MD, USA

**Keywords:** Computable phenotype, Precision medicine, Precision oncology, Cohort selection

## Abstract

Identifying patient cohorts meeting the criteria of specific phenotypes is essential in biomedicine and particularly timely in precision medicine. Many research groups deliver pipelines that automatically retrieve and analyze data elements from one or more sources to automate this task and deliver high-performing computable phenotypes. We applied a systematic approach based on the Preferred Reporting Items for Systematic Reviews and Meta-Analyses guidelines to conduct a thorough scoping review on computable clinical phenotyping. Five databases were searched using a query that combined the concepts of automation, clinical context, and phenotyping. Subsequently, four reviewers screened 7960 records (after removing over 4000 duplicates) and selected 139 that satisfied the inclusion criteria. This dataset was analyzed to extract information on target use cases, data-related topics, phenotyping methodologies, evaluation strategies, and portability of developed solutions. Most studies supported patient cohort selection without discussing the application to specific use cases, such as precision medicine. Electronic Health Records were the primary source in 87.1 % (N = 121) of all studies, and International Classification of Diseases codes were heavily used in 55.4 % (N = 77) of all studies, however, only 25.9 % (N = 36) of the records described compliance with a common data model. In terms of the presented methods, traditional Machine Learning (ML) was the dominant method, often combined with natural language processing and other approaches, while external validation and portability of computable phenotypes were pursued in many cases. These findings revealed that defining target use cases precisely, moving away from sole ML strategies, and evaluating the proposed solutions in the real setting are essential opportunities for future work. There is also momentum and an emerging need for computable phenotyping to support clinical and epidemiological research and precision medicine.

## 1. Introduction

A common challenge in biomedicine is accurately recognizing cases with particular characteristics to support various tasks ranging from patient enrollment in clinical trials to informing treatment plans to safety signal detection in postmarket reports. Historically, clinical epidemiology and clinical research consistently handled some of these tasks by adapting dedicated case definitions that combine standard clinical criteria to establish particular diagnoses [1]. On the other hand, genomics and other biomedical domains mostly refer to the correct recognition of “phenotypes”, a term that comes from the Greek compound word “φαινότυπος”, composed of “φαίνω” (phaino, “to show, to appear”) and “τύπ_ο_ς_”_ (túpos, “mark, impression, type”), which have been defined as “an individual’s observable traits determined by genotypic and environmental factors” [2]. In the clinical context, “phenotype” primarily refers to a patient’s normal morphological, physiological, or behavioral characteristics as well as the deviations from these characteristics occurring in disease status [3]; however, an abnormal phenotype, as the ecphrasis of a person’s genotype, may be associated with genetic alterations. For example, pathogenic variants in the BRCA1 and BRCA2 genes have been associated with hereditary breast, ovarian, and other cancers. Precision medicine and oncology propose to thoroughly examine genetic information and combine it with clinical findings to achieve complete or “deep phenotyping” for a patient and determine the right treatment plan. Earlier studies, first by Tracy [4] and then by Robinson [3], brought the “deep phenotyping” concept into personalized and precision medicine that both target the delivery of the appropriate therapy and care tailored to a patient’s unique personal characteristics. A subtle difference between the two is that personalized medicine may not necessarily evaluate genetic information and also examine other factors (such as an individual’s preferences and social context), while precision medicine, as a more recent approach, builds the patient’s holistic profile using multiple sources (such as next-generation sequencing and proteomics) to reach an accurate diagnosis and determine the appropriate care plan according to established knowledge and active clinical studies [5].

Inspired by the above terms and, likely, the emerging focus on personalized and precision medicine, the research community coined the broader concept of “computable phenotype” (and a few relevant synonym terms) to refer to “the product of using an executable set of algorithms to identify specific, measurable constructs present in patient records [6].” Computable phenotyping essentially translates a set of criteria uniquely characterizing a disease, condition, indication, patient cohort, or clinical event into a query that can be applied to a data source to retrieve cases satisfying these criteria. With the availability of large amounts of electronic healthcare data, traditional clinical epidemiology and research as well as personalized and precision medicine approaches to recognizing cases with particular characteristics have fallen under the computable phenotype umbrella. Such automated algorithms are increasingly developed to process electronic Health Records (EHRs) and assist clinical research, e.g., by improving clinical trial recruitment [7]. However, this is not the only data source used, as phenotyping often requires parallel processing of multiple sources outside EHRs [8]. This demand is highly visible in the precision medicine and oncology fields, where, as discussed above, there is a need to precisely capture an individual’s characteristics (genomic, clinical, and other) and combine them with external knowledge and other data elements when building computable deep phenotyping approaches, which can, for example, inform treatment plans for a patient. Given the complexity of information evaluated in precision medicine, the increasing complexity of clinical outcomes used in epidemiological and clinical research, and the rate structured and unstructured data are created, advanced and sophisticated approaches are necessary for building efficient computable phenotypes.

Traditionally, researchers relied on expert knowledge of diagnostic codes to represent clinical concepts, mostly the International Classification for Diseases (ICD) codes [9], and a few structured fields, primarily demographics and laboratory tests, to select records from administrative claims and clinical data. This approach typically required validating the code-based algorithm against a set of cases classified by human experts. Sometimes the code-based algorithms did not perform well, leaving a gap for the efficient use of these methods. The sole use of billing codes that may partially (or incorrectly) capture a patient’s status and the absence of vital information has raised some reasonable criticism on the accuracy of (over)simplified approaches. Incorporating more data, such as unstructured clinical notes, and integrating it with additional information using natural language processing (NLP) and other techniques may augment the reliability of automated phenotyping. However, some of these techniques must handle significant data quality issues, including inconsistency, inaccuracy, incompleteness, and missingness that jeopardize the secondary use of EHRs and other data sources [10,11]. Even with improvements in data and algorithms, efficient validation and measurement of algorithm performance remain difficult. This challenge is particularly apparent in precision medicine and other areas that require high-quality phenotypic data to feed advanced pipelines.

Many researchers and interdisciplinary consortia have invested effort and resources in computable phenotyping, such as the Electronic Medical Records and Genomics (eMERGE) program that was launched around 2006 to investigate genetic diseases by identifying large numbers of patients in case groups and control groups with medical and genetic covariates [12]. The Phenotype KnowledgeBase (PheKB) is one of the eMERGE achievements that may support the collaborative development, validation, and sharing of electronic phenotypes across multiple institutions [13]. Moreover, the Observational Health Data Sciences and Informatics (OHDSI) program [14], the National Patient-Centered Clinical Research Network (PCORnet) [15,16], and the US Food and Drug Administration Sentinel System [17,18] have promoted the reusability of phenotype definitions across participating clinical sites by introducing Common Data Models (CDMs) [19–21] and building dedicated software toolkits to support relevant tasks [22–28]. Importantly, evaluation strategies and validation expectations significantly differ across use cases. For example, for applications in drug safety assessment, computable phenotype algorithms are still expected to be validated against human-expert evaluated medical records [18], while in precision medicine applications, validation might be driven by the association of genetic characteristics with clinical variables in a computable phenotype algorithm, without human expert review [29].

Our group has been building and evaluating automated approaches to identify cases related to specific conditions in EHR and postmarket data [30,31]. We currently focus on defining a decision-support phenotyping framework in precision oncology and developing the corresponding automated solutions. To best achieve this objective, we initiated this line of work by conducting an extensive scoping review of the literature on the development and automated application of computable phenotyping in the clinical domain, however, without restricting our search to precision medicine. This paper presents the use cases, detailed data maps, computable phenotyping methods, evaluation strategies, and emerging trends with opportunities for future work that have all been analyzed as part of this review.

**Table:** 

Statement of significance	
Problem or Issue	Efficient computable phenotyping is required to support several use cases in biomedicine, especially precision medicine.
What is Already Known	Computable phenotyping translates specific criteria into queries to automatically recognize patient cases with certain characteristics. Automated phenotyping is in high demand for clinical and epidemiological research, as evidenced by a large amount of published literature.
What this Paper Adds	Computable phenotyping may benefit from clearly defining target use cases and building pipelines containing multiple components. The evaluation of novel approaches in the real setting will open new directions in clinical and epidemiological research as well as precision medicine and oncology that precisely capture an individual’s characteristics (genomic, clinical, and other) and combine them with external knowledge.

## 2. Materials and methods

Our scoping review covered papers describing the development and evaluation of tools, (theoretical) frameworks, software, standards, or intelligent automated identification of patient cases or (clinical) events according to specific criteria. Any publications presenting non-clinical or non-human applications, medical image analysis and classification, limited automation, data-driven approaches with poor evaluation or validation analyses, plain review of existing techniques, or application of phenotype detection without any algorithmic details were beyond the scope of our study. Moreover, articles on single-factor phenotyping, i.e., patient identification based on a single parameter, such as disease codes or laboratory tests, were considered of limited interest to deserve a closer look. We also excluded traditional machine learning (ML) studies that evaluated ML models on specific feature spaces without involving other automated techniques in the phenotyping pipeline. Such approaches are standard, and their detailed examination would not significantly contribute to our topic. This analysis would also be redundant as two recent reviews focused on phenotyping involving traditional ML that mostly used a manually-created feature space to train and test several non-generalizable ML models for case classification [32,33].

**Fig. 1.**
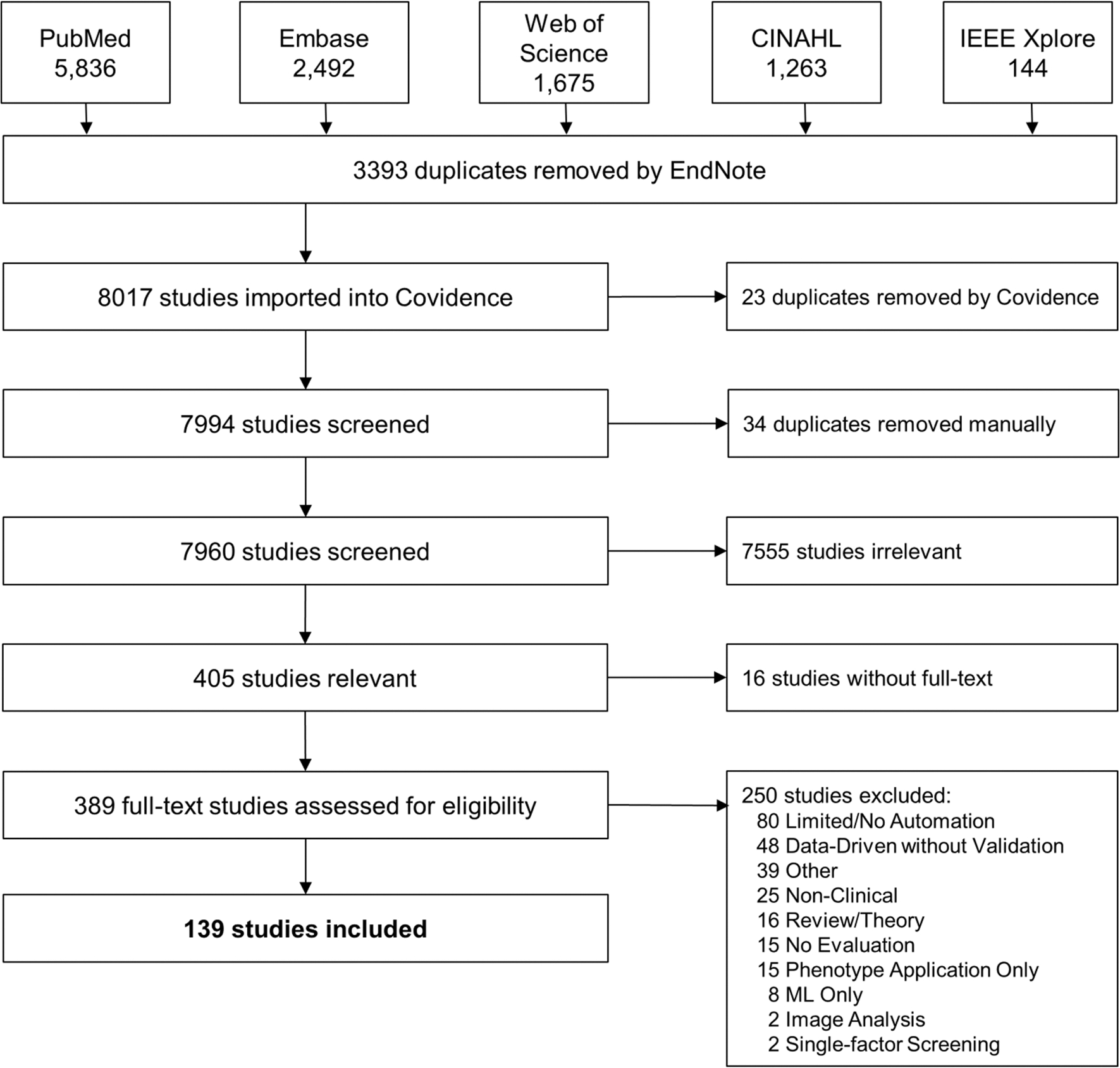
The review process and number of records at each step. We started by retrieving > 8,000 records from five digital libraries and ended with 139 studies in the final dataset that were thoroughly reviewed.

We followed the Preferred Reporting Items for Systematic Reviews and Meta-Analyses (PRISMA) guidelines that describe all steps and best practices for conducting literature reviews and presenting the results [34]. Respecting the PRISMA recommendations, we split the work into four steps: (1) publication retrieval and deduplication, (2) title and abstract review (first screening), (3) full-text review (second screening), and (4) information extraction. The detailed process is shown in Fig. 1.

The EndNote reference management tool helped us organize and deduplicate the publications retrieved in the first step, as well as retrieve full text files for the second screening. To manage the screening and information extraction steps, we used the Covidence web-based tool, which is equipped with advanced capabilities to support systematic reviews adhering to PRISMA guidelines [35]. In particular, Covidence allows for importing citations from various reference managers and deduplicating these citations, storing full-text publications, screening abstracts and full-texts by two or more reviewers and an adjudicator, resolving disagreements, and completing a template-based information collection [36].

In the first step, we collaborated with an experienced librarian from the Welch Medical Library at Johns Hopkins University to define the search query, apply it to several databases, and retrieve all relevant publications. The search query was built iteratively over multiple meetings with the librarian to capture the concepts of “automation”, “clinical context”, and “phenotyping”. Several synonym terms, in the form of words and word roots with wildcards, represented these three concepts using the OR operator, and all three groups of terms were combined using the AND operator to synthesize the following query:

*algorithms* **OR** *causal inference* **OR** *causal discovery* **OR** *causal relationship* **OR** *causal association* **OR** *automation* **OR** algorithm* **OR** automat* **OR** semi-automat* **OR** *computable* **OR** computational

AND

*clinical* **OR** *patient*

AND

*phenotype* **OR** case definition* **OR** phenotyp* **OR** case classificat* **OR** chart abstract* **OR** cohort select*

On February 28–9, 2022, different versions of the structured query were applied to five digital libraries: PubMed, Excerpta Medica dataBASE (Embase), Cumulative Index to Nursing and Allied Health Literature (CINAHL), Institute of Electrical and Electronics Engineers (IEEE) Xplore, and the Web of Science. Each version, included in the Appendix, was used to maximize the search capabilities of the selected libraries and retrieve all relevant peer-reviewed papers published from January 1, 2012, to the search dates. The records obtained from all searches were merged in a single EndNote file and deduplicated using the “Find Duplicates” function. Subsequently, all records were uploaded to a new Covidence project, where a new round of deduplication was executed to prepare the records for the second step of the scoping review.

In the second step, four reviewers (TH, AB, JP, and TB) screened the titles and abstracts of all records in Covidence by applying the criteria summarized below (Table 1). An initial set of 50 records was randomly selected from the included entries by the adjudicator (KK) and was evaluated by all four reviewers to familiarize everyone with the tool and test the application of the screening strategy before proceeding with the processing of all titles and abstracts. Disagreements from the initial set were discussed in a group meeting, and the strategy for the first screening was finalized. Then, the adjudicator calculated the number of records for each reviewer and defined this in Covidence under the “Title and Abstract Screening” step. During this first screening, reviewers also tried to identify and mark duplicate entries manually.

After the first screening, the adjudicator exported all records from the “Title and Abstract Screening” in Covidence to EndNote, where the adjudicator used the “Find Full Text” feature to get the PDF files for as many publications as possible. Following that, the adjudicator uploaded the EndNote records with the PDFs to the “Full Text Review” step in Covidence. To obtain the rest of the PDFs, the adjudicator first performed a Google search for the title and author list and then requested what could not be found (or obtained because of access restrictions) through the Welch Medical Library. These files were imported manually to the “Full Text Review” in Covidence. Publications that were impossible to find the full text had to be excluded from further processing.

In the second screening, two reviewers (TH, AB) independently reviewed all entries in the “Full Text Review” by assigning the exclusion codes (Table 1) that had been previously added to the Covidence project by the adjudicator (all aligned to the exclusion criteria summarized at the beginning of the section).

The two reviewers focused on the Methodology section but read other sections of the paper, if needed, to decide. As in the first screening, they met with the adjudicator to discuss any challenges and get general guidance. For example, a frequent challenge was around papers describing the association of genes and mutations to specific phenotypes; the adjudicator reminded the reviewers that such papers were beyond the scope of our work. It should also be clarified that, in contrast to the first screening, they both reviewed the same papers and independently voted. Disagreements about whether to include or exclude a paper were resolved by the adjudicator. Disagreements about the specific exclusion code to use for a paper were also resolved by the adjudicator after a more cursory review that involved a quick read and selection of the most appropriate reason between reviewers’ suggestions. The inter-reviewer agreement was calculated both as the percentage of agreements across all records and using Cohen’s kappa [37], which accounts for the probability of chance agreement based on how frequently reviewers chose to include or exclude papers.

In the last step, we collected the information for computable phenotyping in clinical and epidemiological research as well as precision medicine from the papers that satisfied the inclusion criteria. We defined six broad categories and multiple items per category to capture all information of interest. An extensive data extraction template was created in Covidence and contained the following six categories and particular items:

1. Use cases: Information about the research area or setting, the disease (s) or condition(s) examined, whether specific phenotype algorithms vs. generalizable phenotyping methodologies were proposed, and the format of what was presented.
2. Data sources: Information about the data sources, the type of structured and unstructured data, the potential use of other data types, the data availability, the use of existing terminologies or encoding strategies and common data models, the size of data, and the handling of missing data values.
3. Methods: Information about the category of the method (supervised ML, unsupervised ML, semi-supervised ML, not in the ML field), the definition of phenotypic criteria, the feature generation for model training, the potential use of NLP and deep learning, the exploration of causal associations, any multi-site development and validation, and the reuse of existing methods.
4. Evaluation and User Acceptance: Information about the performance of the developed solution over the primary or external data, the metrics and format of any reference standard used, and user satisfaction with the presented performance.
5. Outcomes: Information about the form of a developed computable phenotype (package, platform, other), availability, and access to the source code.
6. Special Topics: Information about strengths and weaknesses, further steps, and other special topics discussed in the included papers.

Although we anticipated that collecting sufficient information for all items in the six categories would be unfeasible, we considered it essential to evaluate all these parameters in order to best recognize the trends and opportunities in computable phenotyping.

## 3. Results

The publication retrieval (step 1) from all databases resulted in 11,410 records, with the most obtained from PubMed (5,836), followed by Embase (2,492), Web of Science (1,675), CINAHL (1,263), and IEEE Xplore (144). These records were imported into an EndNote library and deduplicated using the corresponding functionality that produced 8,017 unique entries. The EndNote file was uploaded to Covidence, and new deduplication was applied to capture any duplicate records missed by EndNote. This deduplication further reduced the dataset size to 7,994 records, which were then screened by the four reviewers (TH, AB, JP, and TB). As it was impossible to have an equal split across the four subsets (2 subsets with 1,999 records and two subsets with 1,998), two reviewers processed one extra record. To expedite the process, no exclusion labels were assigned to the eliminated papers. During the first screening, 34 additional duplicates were found and eliminated. A total of 405 records were included and moved to the third step, i.e., the “Full Text Review”.

To obtain the full texts for the second screening, we first exported the 405 records to EndNote, ran the “Find Full Text” function, and obtained 322 PDFs. Google search returned 62 additional PDFs, and the Welch Medical Library helped us get five more PDFs. A total of 16 records remained without PDF attachments and were excluded from the full text review, leaving 389 records. The EndNote library with the PDFs was uploaded to Covidence under the “Full Text Review” and the additional PDFs (from the Google search and the Welch Medical Library) were added manually.

During the second screening, the 389 full text records were reviewed by the two reviewers (TH, AB), who decided to include the paper or assigned one of the exclusion codes listed in Table 1. The most frequent exclusion reason was “Limited/No Automation” (80 studies), followed by “Data-Driven without Validation” (48 studies), “Other” (39 studies), “Non-Clinical” (25 studies), “Review/Theory” (16 studies), “Phenotype Application Only” (15 studies), “No Evaluation” (15 studies), “ML Only” (8 studies), “Image Analysis” (2 studies), and “Single-factor Screening” (2 studies). Of note, the comments that reviewers attached when applying the “Other” exclusion reason indicated that 19 of those 39 did not include a full-text paper: the PDF just contained a version of the abstract. The general agreement of the reviewers on Include vs. Exclude was 63.0 % with a Cohen’s kappa value of 0.27. Their exact agreement on the specific Exclude code used was 42.7 %. A total of 144 records were adjudicated for disagreements on whether to include, with the adjudicator deciding to Include 29 of them. A further 78 records were adjudicated because of disagreements on the exclusion code that should be applied. The final set for the information extraction step included 139 papers.

The citations for all the deduplicated records reviewed in the “Title and Abstract Review” (N = 7,960), the “Full Text Review” (N = 389), and the “Information Extraction” (N = 139) steps can be found as Supplementary Material in three separate files.

In the information extraction step, the 139 full-text papers were processed by one of the reviewers (TH) to complete as many items as possible in the six categories defined above (use case, data set, methods, outcome, evaluation, and specific topics). It was particularly challenging to collect information for certain items. For example, only 8.6 % (N = 12) of the studies discussed the handling of missing values in the elements synthesizing a computable phenotype, and only 31.7 % (N = 44) mentioned whether an external validation of the proposed method had been conducted. On the other hand, it was easier to identify the data sources used in most papers (87.8 %; N = 122). Acknowledging the skewed distribution in the collected information, we present the main findings from our “Full Text Review” in the following sections.

### 3.1. Use cases

The top investigated conditions were cancer [29,38,39], diabetes mellitus [40–42], heart failure [43–45], systemic lupus erythematosus [46–48], and acute respiratory distress syndrome [49–51]. More than half of the studies focused on diagnosis classification (N =88) to identify patients suffering from typical (N = 84), such as diabetes, and rare (N = 4), such as inborn errors of metabolism, conditions. We also found publications describing (deep) phenotyping work in precision medicine [29,52–54] and personalized medicine [39]. We strictly determined that the precision medicine label would be assigned to studies using genomic data, while the personalized medicine label would be attached to publication records demonstrating the association of a patient or groups of patients sharing the same phenotypic characteristics (not necessarily including genomics) with treatment options. Otherwise important publications that used phenotyping to support subsequent genomic analysis, such as the Kho et al. work [41], or claimed applicability to personalized medicine without demonstrating it, such as the Wirbka et al. study [55], were moved into one of the other categories. Patient stratification, i.e., identifying clusters of patients with the same characteristics after selecting a larger cohort (N = 11 studies), clinical trial screening (N = 6 studies), and medical product adverse event detection (N = 5 studies) were the other use cases supported by the proposed computable phenotypes. The remaining studies did not target specific use cases but rather described supporting methods or technologies (N = 8), such as building a data warehouse [56] and auto-selecting clinical features from publicly available clinical sources to support phenotyping [57], summarized the contribution of major projects (N = 2), discussed evaluation analyses of existing algorithms (N =5), or presented methods built for specific themes in Open Challenges (N = 9).

The type of use case affects the selected methodology or development strategy drastically. For example, Callahan et al. developed a search engine to select cohorts from longitudinal patient records, which falls under the diagnosis classification focusing on temporal data. This engine relied on a new temporal query language (built by the investigators) and not the standard Structured Query Language (SQL) that may not easily and efficiently support the execution of temporal queries [23]. Lasko et al. also incorporated temporal information into their computational engine by transforming raw data into a continuous longitudinal probability density to feed an unsupervised learning process [58]. In a different study for diagnosis classification, Murray et al. argued that an efficient phenotyping system should be able to adjust for clinical uncertainty, which is particularly common, and proposed a probabilistic model adaptable to various clinical needs [47]. We also observed that performance expectations varied across use cases. High sensitivity is often a priority in adverse drug event detection, as detecting all actual cases is critical [59]. On the other hand, genotype and phenotype relationships must be identified with high specificity in precision medicine [41].

### 3.2. Data-related topics

**Fig. 2.**
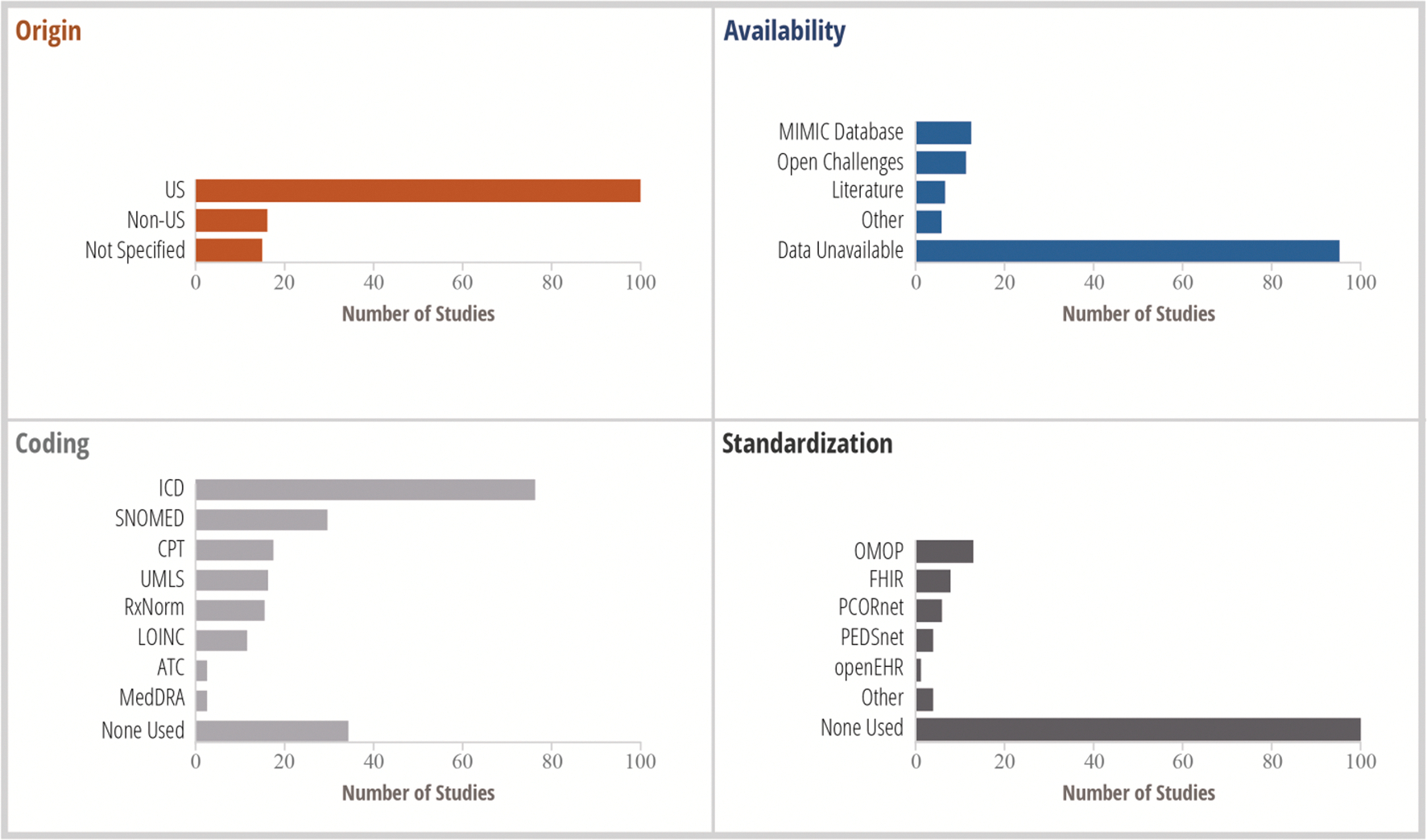
Key parameters characterizing the datasets used. The distribution of publications in the final dataset based on four parameters: origin (indicates whether data was generated in the US or not), availability (shows the source of freely available data), coding (includes standard terminologies used), and standardization (lists common data models used). Abbreviations: US: United States; MIMIC: Medical Information Mart for Intensive Care; ICD: International Classification of Diseases; SNOMED: Systematized Nomenclature of Medicine; CPT: Current Procedural Terminology; UMLS: Unified Medical Language System; RxNorm: standard terminology for medications marketed in the US; LOINC: Logical Observation Identifiers Names and Codes; ATC: Anatomical Therapeutic Chemical; MedDRA: Medical Dictionary for Regulatory Activities; OMOP: Observational Medical Outcomes Partnership; FHIR: Fast Healthcare Interoperability Resources; PCORnet: Patient-Centered Clinical Research network; PEDSnet: Pediatric-Specific network; openEHR: open Electronic Health Record.

Fig. 2 shows the publication distribution when grouped by data origin, availability, coding, and standardization. Almost all studies (N = 137) reported using data for constructing, testing, or validating computable phenotypes and supporting technologies or theoretical frameworks. Most data sources were of US origin and unavailable to the research community. The Medical Information Mart for Intensive Care (MIMIC) database, which contains de-identified information for patients admitted to the intensive care unit at a tertiary hospital [60], and Open Challenge data (from the i2b2 and n2b2 series) were the most highly-used publicly available data (13 and 12 publications, respectively). We also observed that 72.7 % (N = 101) of the selected publications reported using codes from at least one (some combined more) of the common terminologies. As expected, ICD (N = 77), followed by SNOMED (N = 30), was the most frequently used. On the other hand, existing Common Data Models (CDMs) were infrequently used for phenotyping tasks, as indicated by the relevant mentions in 25.9 % (N = 36) of the records in the final set. In addition, seven studies developed or utilized tools complying with the Fast Healthcare Interoperability Resources (FHIR) to support part of or an entire pipeline, such as the SemEHR [61], the NLP2FHIR [62], and the CQL4NLP [63] tools.

#### Data sources and types

**Fig. 3.**
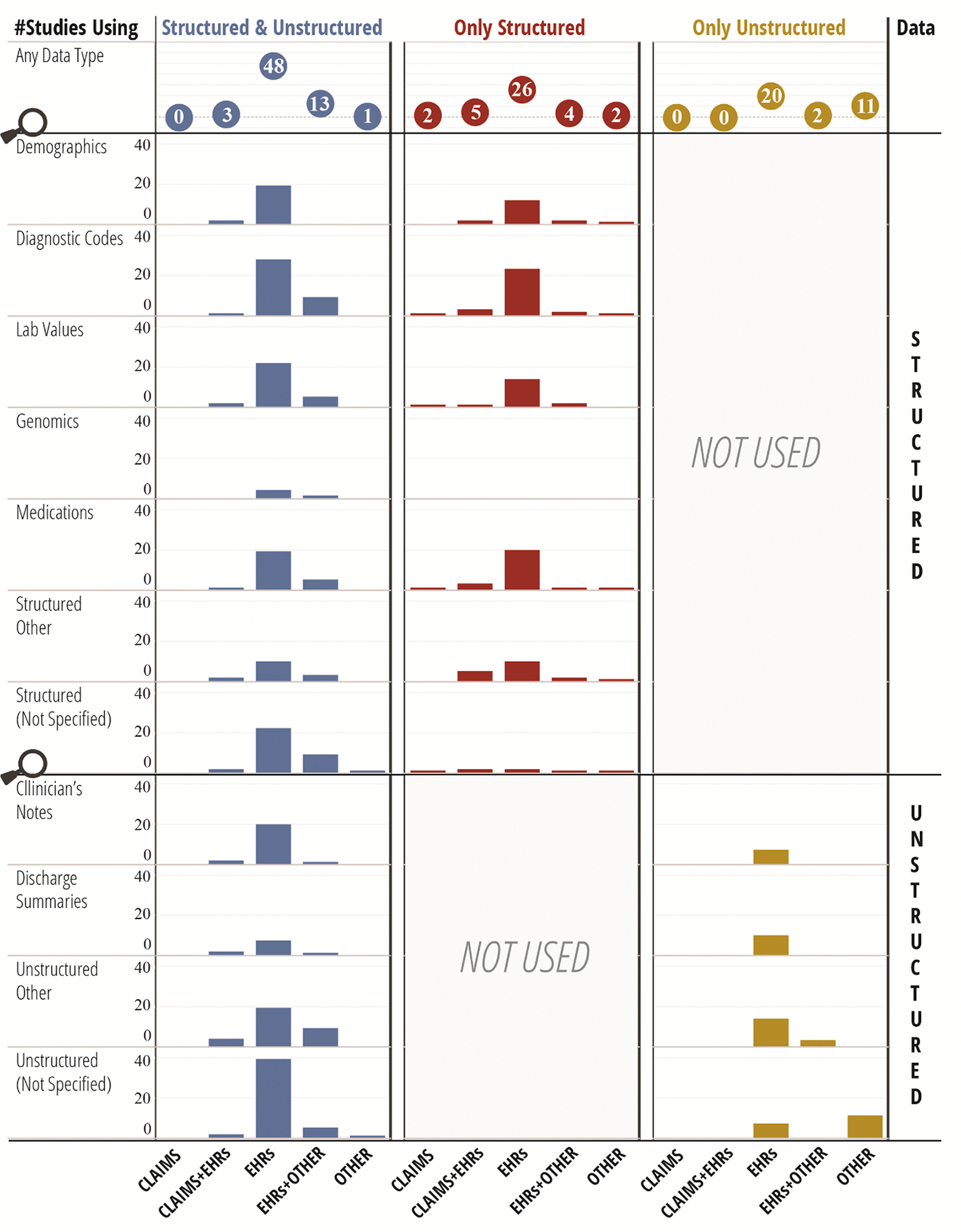
A snapshot of all data sources and types. All studies were split into three major categories. First, studies that analyzed both structured and unstructured data (N = 65; in blue). Second, studies that relied on structured data only (N = 39; in red). Third, studies that analyzed unstructured data only (N = 33; in brown). Two studies did not report any use of data and are not shown. Electronic Health Records (EHRs) were the primary data source used either alone (N = 94) or in combination with claims (N = 8) or other data sources (N = 19) across all three categories. Other data sources included synthetic, postmarket, literature, social media, medical knowledge (e.g., in the form of case definitions), and surveys. Demographics, diagnoses, lab values, and medications were the most used structured data types. Other structured data types included clinical covariates, comorbidities, encounters, vital signs, procedures, progression times, referral Indicators, and synthetic types. The clinician’s notes and discharge summaries were primarily analyzed in terms of the structured data types. Other unstructured data types included admission notes, encounter notes, chief complaint notes, clinical correspondence, consultation notes and letters, progress notes, echo reports, emergency department notes, family records, operative notes, radiology reports, publications, and social media free-text descriptions. (For interpretation of the references to colour in this figure legend, the reader is referred to the web version of this article.)

Nearly half of the studies (46.8 %; N = 65) used both structured and unstructured data to create and test computable phenotype algorithms, while 28.1 % (N = 39) and 23.7 % (N = 33) of the publications reported the sole use of structured and unstructured information, respectively (Fig. 3). Diagnostic codes, lab values, demographics, and medications were the most frequently used structured data types. For unstructured data, clinician’s notes (N =30 studies) and discharge summaries (N =20 studies) were mainly processed to support automated phenotyping. EHRs were the primary source in 87.1 % (n = 121) of the studies, either alone (N = 94) or in combination with claims (N = 8) or other data sources (N = 19). These other data sources included synthetic, postmarket, literature, social media, survey data, and external knowledge in the form of case definitions and ontologies. It should also be noted that genomic data were part of the EHR clinical records in 19 studies only. The remaining studies relied solely on claims data (N = 2) or other sources (N = 14), which included synthetic, postmarket, and non-clinical research data. Two papers did not report using any data and are not included in Fig. 3 [12,13]. It should be clarified, though, that both studies described the critical work conducted by the eMERGE program in computable phenotyping and were relevant to our review.

For the papers reporting on it, adding unstructured data to phenotyping approaches that had relied on structured data did not consistently result in performance improvements. Liao et al. initially developed a coronary artery disease screening algorithm, including parameters that only received values from structured data fields [64]. When they applied NLP to retrieve disease mentions from the clinical notes and map them to broader concepts, the algorithm’s sensitivity was improved in all three patient cohorts analyzed. On the other hand, Moldwin et al. found that adding textual features to structured data algorithms built for 172 clinical phenotypes improved the performance of 51 phenotypes only [65]. No statistically significant change was observed for 120 phenotypes, while the performance was decreased for one phenotype.

#### Data missingness

Missing data is a common challenge in biomedicine and may considerably affect computable phenotyping. Interestingly, only a few publications (N = 12) described any simple or advanced methods to handle data missingness. Two studies added an indicator to missing values [66] and downgraded the phenotyping label assigned by the algorithm to probable or possible [47]. Most groups deleted records with missing data [52,67–69] or imputed the missing values [70–74]. Imputation involved standard techniques (default value assigned by experts [70], Markov Chain Monte Carlo multiple imputation [72], and matrix completion [74]) or was handled by the phenotyping models themselves as described in three studies [71,73,75]. Li et al. developed a multi-view Bayesian phenotyping model that predicted missing diagnostic codes and values based on latent associations in sparse EHR data [73]. Similarly, Hubbard et al. introduced a Bayesian latent phenotyping method to handle missing not-at-random patterns in EHR data [71]. The third study by Zhang et al. completed missing information for a patient visit by inferring it from neighboring (previous or next) visits [75]. For completeness, we should cite eight studies that acknowledged the need to deal with the missingness issue but did not report any relevant strategies [76–80], did not have missing values in the selected dataset [38], and took no actions by either stating that missingness in general “accounts for a minority of patients in the database” [43] or being unable to propose any strategies to correct the otherwise recognized limitation [53].

### 3.3. Computable phenotyping methods

**Fig. 4.**
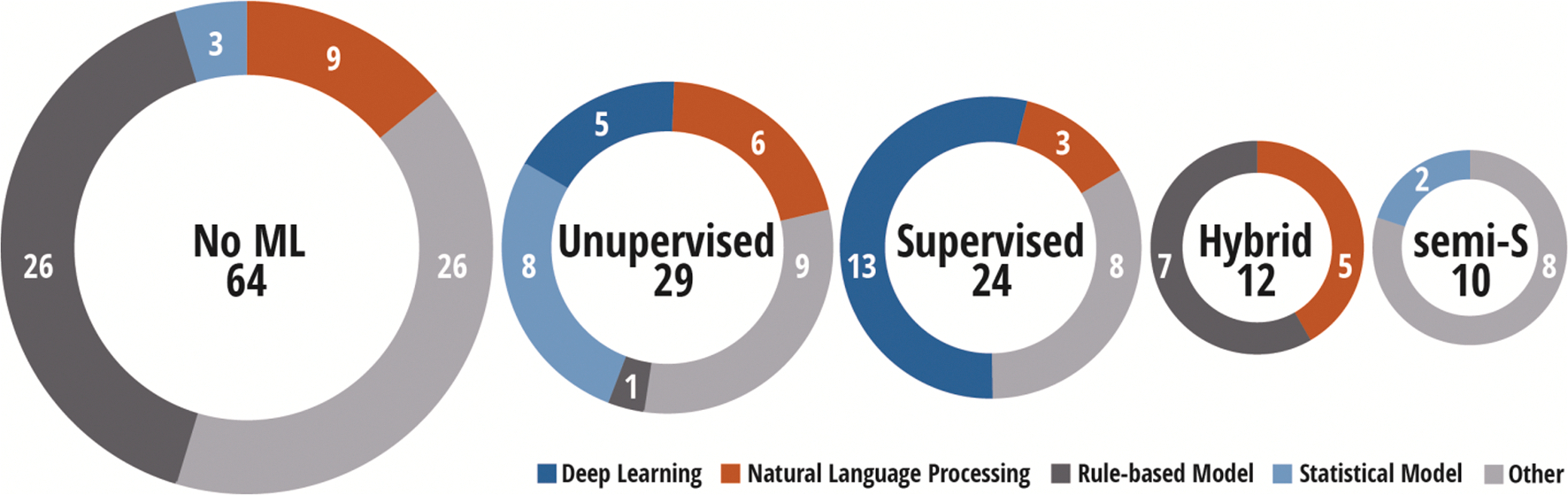
Computable phenotyping methods. Machine learning (ML) is not shown as a separate core approach in the donut charts; it is self-explained that all ML-related categories (Unsupervised, Supervised, Hybrid, and Semi-supervised) include an ML component. More than half of the studies (54 %) used an ML component (deep learning or not) and combined it with natural language processing (NLP), rule-based, or statistical models. These types of models were additionally used in the “No ML” remaining studies (46 %) alone or combined. Abbreviations: ML: Machine Learning; semi-S: semi-supervised.

We evaluated the methods that supported the phenotyping tasks in each study and split them into five broad categories based on the core approach used: supervised ML, semi-supervised ML, unsupervised ML, hybrid ML, and no ML (17.3 %, 7.2 %, 20.9 %, 8.6 %, and 46.0 %, respectively, of the studies; see Fig. 4). Although we excluded papers solely relying on traditional ML, we included records (N = 75) that presented it as the core approach but combined it with other methods, as shown in Fig. 4. The “Hybrid ML” category also contained papers that equally employed some ML and other methods (rule-based or NLP), so it was hard to draw a line and determine a single approach. All remaining papers were placed in the “No ML” category. Rule-based models, NLP, deep learning, and statistical models were the other approaches (in 33, 23, 18, and 13 studies, respectively; Fig. 4) used alone or in combination with ML and other methods, such as anchor learning [22,81], noisy labeling [22,47], and transfer learning [82].

A common denominator in most studies that pursued the identification of specific phenotypes was the initial definition of the problem as binary, i.e., absence vs. presence of the phenotype in question. Only very few deviated from the norm and considered more than two outcomes, such as the Esteban et al. work that categorized patients for their diabetic status using three classes (diabetic, not diabetic, and inconclusive) and practically attempted to capture the inability of automated approaches to assign a strict Boolean label to a patient case reliably [40].

#### Machine learning methods

The most frequently used ML models across all ML-based categories were Random Forests (RF), Naive Bayes, and Support Vector Machines (SVMs), always combined with other methods in the algorithmic pipeline. A few characteristic examples could be mentioned here. In a supervised ML study, Stemerman et al. initially constructed an NLP pipeline to generate and select the features that supported the training and testing of their ML algorithms [83]. A notable unsupervised ML work relied on a knowledge base of biomedical entities to develop an approach for patient stratification and drug repositioning [39]. Semi-supervised ML employed statistical models and other methods along with ML to support specific tasks, such as in the Halpern et al. work that applied anchor-based learning to achieve high positive predictive value and make phenotypic predictions independent of anchors’ presence in the labeled data [81].

#### Rule-based methods

Rule-based models generally incorporate rules and logical constraints based on experts’ clinical judgment, clinical guidelines, previous studies, and, rarely, automated generation processes [84]. In our review, we found representative examples for the first three categories. First, Actkins et al. proposed three different rule-based algorithms for identifying patients with polycystic ovary syndrome based on experts’ clinical judgment [67]. Second, Peer et al. developed a computable phenotype that incorporated existing guidelines and criteria from case definitions to identify asthmatic children and determine the disease severity [79]. Third, Shrephred et al. determined the optimal set of ICD codes for spinal cord injury based on information they retrieved from published work [85].

#### Natural language processing methods

The identified NLP methods either converted unstructured into structured-like data units to feed the actual ML and other classifiers or analyzed the semantics and handled the phenotyping task. Sung et al. demonstrated both uses in their work [68]. Initially, they used the SciSpacy library to parse the unstructured text and prepare it for an RF, SVM, or Elastic Net classifier. Subsequently, they fine-tuned the clinical Bidirectional Encoder Representations from Transformers (BERT) model [86] and applied it to the same data. We should also highlight the Lin et al. work that added the DocTimeRel function to an existing NLP tool, the clinical Text Analysis and Knowledge Extraction System (a.k.a.cTAKES) [87], to identify drug-induced liver toxicity in patients with rheumatoid arthritis [88].

#### Deep learning methods

Deep learning is an emerging methodology observed in recent computable phenotyping studies. Some of these studies combined NLP or transfer learning techniques with various deep learning models, such as convolutional neural networks and text-based graph convolutional networks [62,89]. A recent study by Liu et al. further demonstrated how the standardization of unstructured EHR data with the NLP2FHIR pipeline supported several deep learning models (convolutional neural networks, gated recurrent units, and text-based graph convolutional networks) and resulted in better performance than using raw text [62]. Furthermore, a few scholars have proposed statistical models to support phenotyping tasks in a more traditional mode. The selection of probabilistic models that handled data quality issues and likely uncertainty in case classification should be highlighted as a potential solution for use cases that cannot rely on perfect datasets [73,90].

### 3.4. Computable phenotyping evaluation

Most studies (78.4 %; N = 109) used data sources from a single institution to build and, in almost all cases, evaluate phenotyping algorithms, however, some (20.1 %; N = 28) took a step further and included multi-health system data in order to show the robustness of phenotyping algorithms. For example, Kho et al. identified genetic risks for type 2 diabetes by creating rule-based algorithms on the original study site but validated their findings by applying them to data at external sites [41]. The reference standards used for evaluation in 111 studies included expert-annotated datasets (N = 91 studies), open-challenge datasets (N = 16 studies), and synthetic datasets (N = 4 studies). The remaining publications described the human review of algorithmic outputs (N = 24) or no evaluation (N = 4 studies). Accordingly, we recognized three major evaluation strategies: comparing an algorithm’s output with an annotated reference standard, reviewing an algorithm’s output based on human expertise and clinical knowledge, or further analyzing computable phenotyping in a “second-level” validation. This additional analysis occurred in the precision medicine studies that generally explored the association between the EHR-derived phenotypes with genetic features [29,53,54] and any substantial genetic correlations with genome-wide data [52]. Similarly, in the single personalized medicine publication found in our review, medical experts examined whether the top candidate drugs suggested per patient subgroup were the right treatment choice for their condition [39].

#### Evaluation metrics

**Fig. 5.**
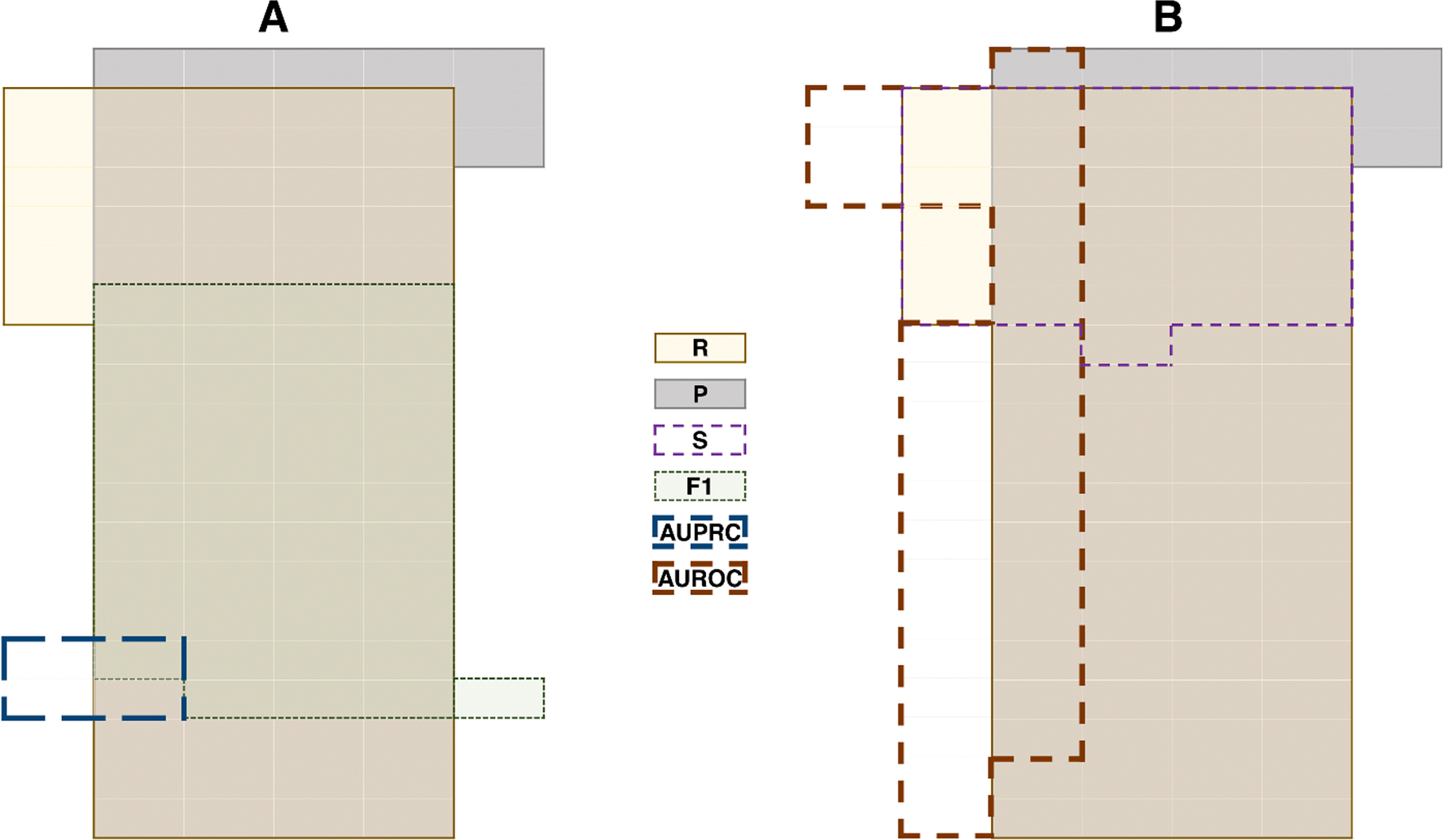
Combination of standard metrics for performance evaluation. A) The first diagram shows how studies combined recall, precision, F1-measure, and AUPRC. Most studies calculated both recall and precision (N = 77), however, some did not combine them by calculating recall (N = 6) or precision (N = 5) only. Moreover, F1-measure was presented in only 43 of the 77 studies that listed recall and precision; one study listed F1-measure without including the other two metrics. Similarly, four papers presented AUPRC but not any of the other metrics. B) The second diagram focuses on precision, recall, specificity, and AUROC. Specificity was always calculated with recall (or sensitivity) in 31 studies and, in most instances, precision (N = 26). AUROC was combined with recall and precision (N = 17), recall and specificity (N = 9), and precision only (N = 1) or was calculated alone (N = 16). *Each cell in a diagram represents one study*. Abbreviations: R: Recall; P: Precision; S: Specification; F1: F1-measure; AUPRC: Area Under the Precision Recall Curve; AUROC: Area Under the Receiver Operating Characteristic Curve.

The selected papers reported evaluation results using primarily the standard metrics of sensitivity (or recall, N = 83), positive predictive value (or precision, N = 82), specificity (N = 31), F1-measure (N = 44), Area Under the Precision Recall Curve (AUPRC, N = 4), and Area Under the Receiver Operating Characteristic Curve (AUROC, N = 37). Interestingly, not all studies that presented both recall and precision (N = 77) calculated the harmonic mean of these two metrics, i.e., the F1-measure (N = 43). Similarly, the AUPRC or AUROC calculations were not always combined with precision and recall or specificity and sensitivity, respectively. Fig. 5 includes two Venn-like diagrams illustrating how studies in the final dataset combined the above standard metrics to demonstrate the performance of their approaches. Detailed numbers can be found in Supplementary Table 1.

A few studies used less common metrics, including the mean average precision or mean reciprocal rank to evaluate the output of their ranking algorithms [76,91] and the C-statistic or Hosmer-Lemeshow goodness-of-fit test to report the reported goodness-of-fit of their models [40,79,92–94]. Moreover, some research groups applied non-standard evaluation approaches, still using some of the same metrics but adding other dimensions. An interesting approach was presented by Halpern et al., who used the AUC metric to investigate the performance of their “learning with anchors” framework at different time points after patients’ arrival at and disposition from the emergency department while new information was being added to their records [81]. No publications reported the routine use of computable phenotypes and end-user satisfaction in the real setting.

### 3.5. Portability of developed solutions

A few studies considered the portability aspect when developing computable phenotypes or releasing tools that support this task. Some tools that develop, evaluate, and practically allow for sharing computable phenotypes across institutions maintaining clinical databases adhering to the same CDM are already making a significant contribution [22–25]. However, the requirement to fit the data to a single CDM is not always functional, as it may require considerable effort to generate the CDM-compliant version of existing clinical data, especially when data elements must be pulled from free texts. Wen et al. presented a promising direction by creating a set of FHIR NLP extensions and NLP rulesets that were then integrated into the NLP2FHIR normalization pipeline and, if assisted by customized dictionaries, they might support information retrieval for phenotyping tasks [63]. We should also acknowledge groups that wrapped their pipelines into publicly available R-statistics packages [95] and Python libraries [96]. However, it should be emphasized that although making resources freely available to the community is paramount, this does not guarantee portability, especially when a considerable effort is required to collect and format the data for these tools to run.

## 4. Discussion

We initially identified a set of over 8,000 publications that were subsequently screened according to specific criteria to generate a final set of 139 studies focusing on computable phenotyping. A common theme in most studies was the selection of patient cohorts using dedicated non-generalizable algorithms without specifically discussing how actual use cases might benefit from the proposed automation. This is interesting, considering that 87 % of the publications in the final set presented approaches, often quite advanced and sophisticated, such as combining NLP with deep learning to process structured and unstructured EHR (live) data for phenotyping purposes. We should acknowledge, though, two types of efforts that might change the momentum and create opportunities for operationalizing computable phenotyping. First, recent studies in precision medicine that explored the association of EHR-derived phenotypes with genetic features or suggested therapies for patient groups with the same characteristics, and, second, studies that pursued the validation of potentially portable and generalizable solutions with multi-source data. Although still too rare to claim that these efforts represent new trends in computable phenotyping, they revealed some opportunities for further investigation and, most importantly, for actual implementation in clinical, epidemiological, or precision medicine research. In the future, the field should leverage ongoing work in other fields, including NLP and language modeling, deep learning, data standardization, and more. Finally, encoding data using standard terminologies, such as ICD and SNOMED, and building pipelines complying with appropriate CDMs, a largely uncommon but vital strategy, would also increase the impact of future work in computable phenotyping.

Our scoping review has three limitations. First, we used only single-reviewer screening for the title and abstract phase, and not all reviewers were involved in the two screening and the information extraction steps, a standard approach applied to systematic reviews. The single reason for this strategy was the availability of resources at the turning points of this nearly 6-month effort. On the other hand, the same adjudicator closely monitored all steps for consistency and guided the four reviewers throughout the process. Second, one might criticize the efficiency of the selected query in retrieving all (or nearly all) the relevant articles from the whole body of biomedical literature. This concern is frequent in systematic reviews, and the study team must weigh all parameters, such as the trade-offs of more or less specific terms, before defining the optimal query. We argue that our search was broad enough and returned a large set of citations that helped us evaluate the trends and opportunities in the field, especially in the context of precision medicine. We acknowledge that the exclusion of “ML only”, “Data-Driven without Validation”, and “Limited/No Automation” to computable phenotype algorithm construction eliminated many papers from this review, but our intent was to identify approaches that automated multiple steps in the phenotyping pipeline and handled complex tasks, often by using hybrid solutions. Third, although all reviewers were trained (before and during the testing phase with the 50 abstracts) and continuously interacted with the adjudicator, their agreement on Include vs. Exclude in the second screening was fair (Cohen’s kappa = 0.27). Similarly, their exact agreement on the specific exclusion code used was 40.9 %. We acknowledge this limitation and believe it was adequately addressed in the adjudication process.

To the best of our knowledge, only a few comprehensive reviews have been conducted on computable phenotyping over the last ten years. An early and highly-cited paper examined automated approaches for cohort identification without discussing any more specific applications [84], a topic extensively discussed in a recent review describing all the use cases we presented above [97]. Both publications listed the same data sources found in our study, with the exception of imaging data, due to our decision to exclude image analysis and classification studies. They also reported the same primary methods and most of the existing NLP tools captured in our exploration, although, due to the timeframe of the review, the first publication did not identify any deep learning methods and only recognized a trend in ML with the rule-based solutions dominating the field [84]. Surprisingly, any reference to hybrid systems was somewhat limited, but they both agreed on the promising nature of these strategies. Regarding challenges and expectations for future work, we observed consensus on the need to build generalizable, interpretable, and efficient solutions. Interpretability was emphasized for the hybrid [84] and the deep learning models [97] that generally lack(ed) clarity on the pipeline architecture and execution of the phenotyping task. Efficiency was criticized by (in)directly referring to approaches’ poor-to-average performance measured with the standard metrics listed above. The less common metrics and non-standard evaluations noted in our review indicate that new use cases with unique characteristics and expectations require different evaluation processes. The early review paper further suggested investing more effort in utilizing or improving terminologies and using administrative codes less without referring to CDMs [84,97].

As NLP is a significant component in phenotypic pipelines, we should cite a third review that explored the application of NLP technologies in precision oncology to generate features required for computable phenotyping and directly support cancer patient care [98]. This study concluded that “NLP technology is not yet ripe for direct patient care except in carefully observed scenarios,” which is in alignment with the other two papers. Interestingly, the early publication commented on the limited use of NLP methods in computable phenotyping at that time for co-reference resolution, temporal analysis, and assertion classification [84]. Although advanced NLP methods and tools are currently the core components in many phenotyping pipelines, it is evident that there are still concerns about their readiness to assist in operationalizing computable phenotypes.

Apart from the main findings discussed above, it is significant to highlight some major trends recognized in our scoping review. An increasing number of recent studies have proposed solutions adhering to CDMs or standards like FHIR, significantly improving the portability of these solutions across different systems compliant with such standards. While our review excluded “ML only” approaches, we identified many deep learning and hybrid ML-based solutions that efficiently handle complex tasks. This might suggest that the field is transitioning to more sophisticated methods to accommodate the availability of more data and data types and the need for subtle distinctions in computable phenotypes. As more unstructured data sources feed the newer algorithms, more NLP components are incorporated. Moreover, patients’ genomic profiles are analyzed in some studies, and although this does not qualify as a trend, we believe that future work will heavily utilize genomics and continue building toward precision medicine.

The recognized trends and findings shed light on some crucial opportunities for future work in computable phenotyping. We need to see more studies that will automate most (if not all) of the steps in the phenotyping pipeline and present generalizable, reproducible, and validated approaches. Approaches to efficient validation remain a concern. At present, except in unusual circumstances where a phenotype can be easily defined with laboratory data [27], the only reliable approach to validation is by testing an algorithm’s performance against a human expert-created dataset. Automated approaches to improving the efficiency of creating such datasets are needed. Association studies, as identified in this review for the precision medicine use case, might work in some situations, but other promising alternatives need to be further developed [99,100]. Along the same lines, standard evaluation metrics, such as recall and precision, are helpful but may not reliably reflect the actual performance of a solution in the target use case, especially when placed into a live setting. Moreover, a few recent studies have moved away from the traditional ML heavily used in earlier years as the panacea to all problems and proposed hybrid phenotyping solutions. This strategy is on the right track as it may efficiently address challenges related to low data quality, processing of unstructured data, and data standardization and harmonization.

Finally, we argue that there is slowly-building momentum toward precision medicine in this space, and we expect it to grow into a primary use case in computable phenotyping. Interestingly, most retrieved studies lacked the “deep phenotyping” aspect that is now vital for delivering the proper care. Future automated phenotyping must incorporate components to capture patients’ molecular characteristics and the related scientific knowledge to assist physicians in defining the most accurate phenotype to support clinical and epidemiological research, identify genetic and other risk factors, and select optimal therapies and clinical trials for their patients by efficiently monitoring drug safety.

## 5. Conclusions

Computable phenotyping is a major area in biomedicine as it may automate the identification of patients with specific characteristics and, thus, support several use cases in clinical and epidemiological research and precision medicine. In this paper, we reviewed previous efforts in computable phenotyping as they were described in approximately 8,000 scientific publications. After applying specific selection criteria, we closely analyzed a subset of 139 publications to retrieve information about use cases, data-related topics, methods, evaluation strategies, and portability of developed solutions. Most studies in this final set presented computable phenotypes that supported patient cohort selection without discussing a specific use case; however, we found efforts focusing on computable phenotyping that supported genomic analysis, clinical trial screening, personalized medicine, and pharmacovigilance. These more focused applications show that automated approaches must support narrower and more demanding new tasks. We also found that, although standard terminologies, such as ICD and SNOMED, were heavily used, only 25.9 % of the publication records reported adherence to a CDM, indicating an unmet need to combine computable phenotyping with complete data standardization strategies. EHRs were the most frequently used data source, either alone or in combination with other data sources, analyzed with powerful NLP components, such as BERT models, incorporated into the phenotyping pipelines. Traditional ML and, more recently, deep learning appeared in more than half of the publications, which, combined with sophisticated NLP of EHR and other data and the integration with external information and knowledge sources, likely sheds some light on what we expect to see in the future. It was promising that validation with multi-source data was pursued in 20.1 % of the cases, but this number should be pushed even higher, as new works aim for the vital goal of delivering highly portable solutions in the future. We contend that biomedicine and precision medicine, in particular, cannot progress without computable phenotyping, and we hope that our study will inspire the blueprint for growth in this area.

## Supplementary Material

Supplementary material

## Figures and Tables

**Table 1:** Exclusion criteria used in the second screening during the “Full Text Review”.

Exclusion Criteria (Full Description)	Exclusion Criteria (Coded)

Application of phenotype only, no development or algorithmic details	Phenotype Application Only
Applied Machine Learning only, with no other techniques	ML Only
Data-specific or data-driven and without external validation	Data-Driven without Validation
No testing or evaluation results of the phenotyping approach	No Evaluation
Image analysis or image classification	Image Analysis
Limited or no automation in applying the phenotype	Limited/No Automation
Not clinical (including gene-phenotype associations)	Non-Clinical
Single-factor screening	Single-Factor screening
Sole review or a description of a method/technique	Review/Theory
Other species, not human	Non-Human
Other	Other

## Data Availability

As mentioned in the main text, the citations for all records reviewed in the “Title and Abstract Review”, the “Full Text Review”, and the “Information Extraction” steps can be found as Supplementary Material in three Research Information System (RIS) files.

## Appendix A

The entries below describe the queries run in the five databases searched for relevant publications. These included PubMed, the Excerpta Medica dataBASE (Embase), the Cumulative Index to Nursing and Allied Health Literature (CINAHL), the Web of Science, and the Institute of Electrical and Electronics Engineers (IEEE) Xplore database.

**Table:**

Database:	PubMed

Date of Search:	2022-02-08
Query:	(“algorithms”[MeSh] OR “causal inference” [tiab] OR “causal discovery”[tiab] OR “causal relationship” [tiab] OR “causal association” [tiab] OR “automation”[MeSh] OR algorithm*[tiab] OR automat*[tiab] OR semi-automat*[tiab] OR computable[tiab] OR computational[tiab]) AND ((clinical[tiab] OR patient[tiab]) AND (“phenotype”[MeSh] OR “case definition*”[tiab] OR phenotyp*[tiab] OR “case classificat*”[tiab] OR “chart abstract*”[tiab] OR “cohort select*”[tiab]))
Notes:	• [MeSH] – search within the assigned Medical Subject Headings (MeSH) terms [tiab] – search within the title and abstract The * character is a wildcard symbol, which allows for different endings of a word Query was restricted to English articles only Query was restricted to publication date between 2012-01-01 and 2022-02-08

**Table:**

Database:	Embase

Date of Search:	2022-02-08
Query:	(’algorithm’/exp OR ’automation’/exp OR algorithm*:ti,ab OR ’causal inference’:ti,ab OR ’causal discovery’:ti,ab OR ’causal relationship’:ti,ab OR ’causal association’:ti,ab OR automat*:ti,ab OR ’semi automat*’:ti,ab OR computable:ti,ab OR computational:ti,ab) AND ((clinical OR patient) NEAR/3 (’phenotype’ OR ’case definition*’ OR phenotyp* OR ’case classificat*’ OR ’chart abstract*’ OR ’cohort select*’)) AND ([2012–2022]/py AND [english]/lim)
Notes:	• /exp – search for specified term and also related terms ti,ab – search within the title and abstract NEAR/3 – search for two words/patterns that occur within three words of each other The * character is a wildcard symbol, which allows for different endings of a word Query was restricted to English articles only Query was restricted to publication date between 2012-01-01 and 2022-02-08

**Table:**

Database:	CINAHL

Date of Search:	2022-02-09
Query:	(MH “Algorithms”) OR (MH “Automation”) OR (algorithm* OR “causal inference” OR “causal discovery” OR “causal relationship” OR “causal association” OR automat* OR semi-automat* OR computable OR computational) AND (clinical OR patient) AND ((MH “Phenotype”) OR “case definition*” OR phenotyp* OR “case classificat*” OR “chart abstract*” OR “cohort select*”)
Notes:	• MH – search within the major or minor subject The * character is a wildcard symbol, which allows for different endings of a word Query was restricted to English articles only Query was restricted to publication date between 2012 and 01-01 and 2022-02-09

**Table:**

Database:	Web of Science

Date of Search:	2022-02-09
Query:	((algorithm* OR “causal inference” OR “causal discovery” OR “causal relationship” OR “causal association” OR “automation” OR automat* OR semi-automat* OR computable OR computational) AND ((clinical OR patient) NEAR/3 (“phenotype” OR “case definition*” OR phenotyp* OR “case classificat*” OR “chart abstract*” OR “cohort select*”)))
Notes:	• NEAR/3 – search for two words/patterns that occur within three words of each other The * character is a wildcard symbol, which allows for different endings of a word Query was restricted to English articles only Query was restricted to publication date between 2012 and 01-01 and 2022-02-09

**Table:**

Database:	IEEE Xplore

Date of Search:	2022-02-09
Query:	(algorithm* OR “causal inference” OR “causal discovery” OR “causal relationship” OR “causal association” OR “automation” OR automat* OR semi-automat* OR computable OR computational) AND ((clinical OR patient) AND (“phenotype” OR “case definition*” OR phenotyp* OR “case classificat*” OR “chart abstract*” OR “cohort select*”))
Notes:	• The * character is a wildcard symbol, which allows for different endings of a word Query was restricted to publication date between 2012 and 01-01 and 2022-02-09

## Appendix B. Supplementary material

Supplementary data to this article can be found online at https://doi.org/10.1016/j.jbi.2023.104335.
